# Associations between the rumen microbiota and carcass merit and meat quality in beef cattle

**DOI:** 10.1007/s00253-024-13126-1

**Published:** 2024-04-06

**Authors:** Devin B. Holman, Katherine E. Gzyl, Haley Scott, Cara Service, Nuria Prieto, Óscar López-Campos

**Affiliations:** Agriculture and Agri-Food Canada, Lacombe Research and Development Centre, 6000 C&E Trail, Lacombe, AB T4L 1W1 Canada

**Keywords:** Rumen microbiome, Bacteria, Marbling, Beef cattle, Meat, Carcass

## Abstract

**Abstract:**

The rumen microbiota is important for energy and nutrient acquisition in cattle, and therefore its composition may also affect carcass merit and meat quality attributes. In this study, we examined the associations between archaeal and bacterial taxa in the rumen microbiota of beef cattle and 12 different attributes, including hot carcass weight (HCW), dressing percentage, ribeye area (REA), intramuscular fat content, marbling score, fat thickness, yield grade, moisture content, purge loss, and shear force. There were significant correlations between the relative abundance of certain archaeal and bacterial genera and these attributes. Notably, *Selenomonas* spp. were positively correlated with live weight and HCW, while also being negatively correlated with purge loss. Members of the *Christensenellaceae* R-7, *Moryella*, and *Prevotella* genera exhibited positive and significant correlations with various attributes, such as dressing percentage and intramuscular fat content. *Ruminococcaceae* UCG-001 was negatively correlated with live weight, HCW, and dressing percentage, while *Acidaminococcus* and *Succinivibrionaceae* UCG-001 were negatively correlated with intramuscular fat content, moisture content, and marbling score. Overall, our findings suggest that specific changes in the rumen microbiota could be a valuable tool to improve beef carcass merit and meat quality attributes. Additional research is required to better understand the relationship between the rumen microbiota and these attributes, with the potential to develop microbiome-targeted strategies for enhancing beef production.

**Key points:**

*• Certain rumen bacteria were associated with carcass merit and meat quality*

*• Moryella was positively correlated with intramuscular fat in beef carcasses*

*• Acidaminococcus spp. was negatively correlated with marbling and intramuscular fat*

**Supplementary Information:**

The online version contains supplementary material available at 10.1007/s00253-024-13126-1.

## Introduction

The rumen microbiome plays a significant role in beef cattle health and production. For example, bacteria in the rumen provide up to 70% of the daily energy requirements of cattle in the form of short-chain fatty acids (SCFAs) such as acetate, butyrate, and propionate through the metabolism of otherwise nondigestible plant carbohydrates such as cellulose (Bergman 1990). The composition of the rumen microbiome is strongly shaped by diet but can also be affected by other factors such as host age, sex, and genetics (Li et al. 2019). Animal performance traits including average daily gain and feed efficiency have also been linked to specific microbial states in the rumen (Lima et al. 2019; Myer et al. 2015).

In the North American beef industry, the value of a carcass is largely determined by the degree of marbling or intramuscular fat due to its strong association with flavor and palatability (Corbin et al. 2015). Similar to the rumen microbiome, marbling score and intramuscular fat content are determined by factors such as genetics, diet, age, management, and environment (Park et al. 2018). Acetate and lactate produced by the bovine rumen microbiome are used as substrates for lipogenesis as is glucose generated through gluconeogenesis from propionate (Pethick et al. 2004), and therefore the rumen microbiome may also influence intramuscular fat content. Indeed, recent studies with relatively small numbers of cattle have linked the rumen microbiome to certain beef quality traits such as marbling (Kim et al. 2020; Krause et al. 2020). In addition to marbling, other characteristics such as hot carcass weight (HCW), dressing percentage, rib eye area (REA), and retail cut yield (RCY) are also important factors in determining overall carcass market value.

Presently, there is only limited information on associations between the rumen microbiota of beef cattle and carcass merit and meat quality. An association between the rumen microbiome and carcass merit and meat quality traits would suggest that it may be possible to manipulate the microbiome to improve these traits and, hence, enhance livestock efficiency and profitability. Therefore, the objective in the present study was to identify bacterial taxa in the rumen microbiota of steers that are associated with various carcass merit and meat quality traits.

## Materials and methods

### Animals and sample collection

Angus × Simmental steers (*n* = 201) from the Agriculture and Agri-Food Canada Lacombe Research and Development Centre (AAFC Lacombe RDC) herd, were used in this study. Full details of the experimental animals and carcass merit methodology have been previously provided by Segura et al. (2023). Briefly, banding was used to castrate all calves at 24 h of age and then two different production systems, calf-fed (spring) and yearling-fed (fall), were used to generate variation in the carcasses that is representative of the North American commercial cattle industry (Canfax 2023; United States Department of Agriculture 2023). Both groups of steers were also weaned at 6 to 7 months of age. The calf-fed steers were transitioned to a high grain diet over 1 to 2 months, and then fed a high-concentrate diet (78% rolled barley and 22% barley silage) for 150 to 180 days. The yearling-fed steers were provided with a backgrounding diet (higher forage content) for 5 to 6 months and then moved to a high-concentrate diet (78% rolled and 22% barley silage) for 100 to 120 days.

The steers of both production systems were subjected to one of two implant regimens: with or without implants. For calf-fed steers receiving an implant (120 mg trenbolone acetate and 24 mg estradiol, Component TE-S, Elanco-Animal Health, Eli Lilly Canada Inc., Toronto, ON, Canada), this was done before feedlot entry. For yearling-fed steers that were implanted, this was done during the backgrounding period at 90-to-100-day intervals (200 mg progesterone and 20 mg estradiol benzoate, Component E-S, Elanco) and also prior to feedlot entry (120 mg trenbolone acetate and 24 mg estradiol, Component TE-S). When the animals reached market weight, they were slaughtered and processed at the AAFC Lacombe RDC abattoir. The cattle had ad libitum access to water and were slaughtered within 3 h using a captive bolt and exsanguination.

### Carcass merit and rumen samples

Following slaughter, the carcasses were dressed, split, and weighed (HCW in kg). The ratio of HCW to live weight (dressing percentage = HCW × 100/slaughter weight) was used to calculate the hot dressing percentage. After evisceration, an incision was made in the rumen with a sterile knife and a medium sized opening (approximately 30 cm wide) was created to expose the rumen contents. A mixture of solid and fluid rumen contents was obtained from five different areas in the rumen using a sterile glove and a sterile 50-ml centrifuge tube. The samples were immediately stored at −80 °C until further analyses.

The carcasses were chilled at 2 °C for 72 h, and the left side of each carcass was knife-ribbed between the 12th and 13th ribs as per Segura et al. (2023). The carcasses were exposed to atmospheric oxygen for 20 min and evaluated by a Canadian Beef Grading Agency certified grader. The grading assessments included the measurement of backfat thickness at the three-quarters position from the spinous process (in mm), the REA in square centimeters of the longissimus thoracis (LT), and a subjective marbling score, using as reference points the beef marbling pictorial standards from the United States Department of Agriculture (1989). The RCY percentage was calculated as detailed in Segura et al. (2021).

### Meat quality analyses

At 3-d post-mortem, the LT was collected, weighed, vacuum packaged, and aged for an additional 3 d. After the aging period, the LT was unpackaged and weighed to calculate the purge loss. From each ribeye, the two caudal-most 2.5-cm thick steaks were then removed, and the first steak was designated for shear force analyses whereas the second steak was ground with a BX3 Blixir (Robot-Coupe USA Inc., Ridgeland, MS, USA) and then frozen at −20 °C for further proximate analyses. Subsequently, the first caudal-most steak was cooked using a 200 °C ED30B Garland grill (Condon Barr Food Equipment Ltd., Edmonton, AB, Canada) to attain a 71 °C internal temperature. Further cooking was prevented by immediately placing the steaks into polyethylene bags, which were then sealed, immersed in a water bath with ice for 20 min, and transferred to a 2 °C cooler for 24 h. Six 1.90-cm diameter cores were cut parallel to the longitudinal orientation of the muscle fibers and then sheared once perpendicular to the muscle fibers using a TA-XT Plus Texture Analyzer with a Warner-Bratzler shear device attached (Texture Technologies Corp., Hamilton, MA, USA). The crosshead speed was 200 mm/min and the load cell was 50 kg. The average peak force of all six cores was used to calculate shear force (kg). After thawing for 24 h at 4 °C, a 50-g subsample of the second steak was assessed for fat and moisture content following the method 2008.06 of the AOAC (Leffler et al. 2008) and using a Smart Turbo Moisture Analyzer 907990 and a Smart Trac Fat Analyzer 907955 (CEM Corporation, Matthews, NC, USA).

### Rumen DNA extraction

DNA was extracted from the rumen samples with a DNeasy PowerSoil Pro Kit (Qiagen, Toronto, ON, Canada) according to manufacturer’s instructions and included a beat-beating step in a FastPrep-24 (MP Biomedicals, Solon, OH, USA) at 4.0 m/s for 45 s. The DNA was quantified using the Qubit dsDNA HS assay kit (Thermo Fisher Scientific, Mississauga, ON, Canada).

### 16S rRNA gene sequencing

To characterize the rumen microbiota, the modified 515F (5′-GTGYCAGCMGCCGCGG TAA-3′) and 806R (5′-GGACTACNVGGGTWTC TAAT-3′) (Walters et al. 2016) primers were used to amplify the V4 region of the archaeal and bacterial 16S rRNA gene in a two-step PCR. The FastStart High Fidelity PCR System (Roche, Montreal, QC, Canada), 0.2 mM dNTPs, and 5% dimethyl sulfoxide (DMSO) in a total volume of 24 μl was used in the first PCR on a SimpliAmp thermal cycler (Thermo Fisher Scientific, Mississauga, ON, Canada). This included an initial denaturation for 2 min at 94 °C, followed by 26 cycles of 94 °C for 30 s, 58 °C for 30 s, and 72 °C for 30 s, and a final extension for 7 min at 72 °C. These amplicons along with sequencing adapters and dual indexes (Standard BioTools Inc, San Francisco, CA, USA) were included in a second PCR step using an initial denaturation for 10 min at 95 °C, followed by 15 cycles of 95 °C for 15 s, 60 °C for 30 s, and 72 °C for 60 s, and a final extension for 3 min at 72 °C. Agarose (2%) gel electrophoresis was used to verify amplification for both PCR steps.

Amplicons were quantified prior to pooling in equimolar concentrations using a Quant-iT PicoGreen dsDNA Assay Kit (Thermo Fisher Scientific, Mississauga, ON, Canada). sparQ PureMag beads (QuantaBio, Beverly, MA, USA) were used to purify the pooled libraries, and the libraries were quantified using the Kapa Illumina GA with Revised Primers-SYBR Fast Universal kit (Kapa Biosystems, Wilmington, MA, USA). The average fragment length was assessed with a LabChip GX instrument (PerkinElmer, Waltham, MA, USA). A MiSeq Reagent Kit v2 (500 cycles; Illumina, Inc., San Diego, CA, USA) and an Illumina MiSeq instrument were then used to sequence the 16S rRNA gene libraries.

### 16S rRNA gene sequence analysis

Reads were processed and trimmed with Cutadapt v.4.1 (Martin 2011) to remove adapter and primer sequences and discard reads shorter than 215 bp. Amplicon sequence variants (ASVs) were resolved with DADA2 v. 1.24.0 (Callahan et al. 2016) in R v. 4.2.1. Briefly, forward and reverse reads were trimmed to 200 bp, sequencing error rates were learned and dereplicated, and true sequence variants were inferred prior to merging the forward and reverse reads with a minimum overlap of 75 bp. Chimeras were removed prior to assigning taxonomy with the SILVA database v.138.1 (Quast et al. 2013) and the naïve Bayesian classifier (Wang et al. 2007). ASVs that were classified as chloroplasts, mitochondria, or eukaryotes were removed prior to analyses. There were also four ASVs classified as *Escherichia*-*Shigella*, *Bacteroides*, [*Ruminococcus*] *torques* group, and *Nitrospira* that were more abundant in the negative extraction controls and therefore removed. The samples were randomly subsampled to 18,075 reads, and Phyloseq v.1.40 (McMurdie and Holmes 2013) and vegan v.2.6.4 (Oksanen et al. 2013) were used in R to calculate alpha diversity metrics as well as Bray-Curtis dissimilarities. The default parameters were used in all bioinformatics software packages unless otherwise stated.

### Statistical analysis

Pearson correlation coefficients were calculated in R to determine associations between carcass merit and meat quality traits and genera with a relative abundance greater than 0.1%. The Benjamini-Hochberg procedure was used to correct *P* values for multiple comparisons. Permutational multivariate analysis of variance (PERMANOVA) and the Bray-Curtis dissimilarities were used to assess the association of carcass merit and meat quality traits with the rumen microbial community structure. The envfit function in vegan with 10,000 permutations was used to fit the carcass merit and meat quality traits as well as the ten relatively most abundant archaeal and bacterial genera to the non-metric multidimensional scaling (NMDS) ordinations of the Bray–Curtis dissimilarities. Differentially abundant genera in the rumen microbiota between cattle whose carcasses had the 50 highest and 50 lowest marbling scores were identified using MaAsLin2 v. 1.12.0 (Mallick et al. 2021) in R. The Mann-Whitney *U* test was used to compare the carcass merit and meat quality traits between the carcasses with the 50 highest and 50 lowest marbling scores.

## Results

### Descriptive statistics of the cattle and carcasses

The average animal live weights at slaughter and the carcass characteristics in terms of HCW, REA, RCY, and marbling score were representative of commercial beef carcasses in North America (Canfax 2023) (Table 1) as well as quality and yield grades (Canadian Beef Grading Agency 2023) (data not shown).

**Table 1:** Carcass merit and meat quality traits of the steers (*n* = 201). Values represent the mean ± standard error

Attribute	Value
Live weight (kg)	739.2 ± 3.5
Hot carcass weight (kg)	437.4 ± 2.4
Hot dressing (%)	59.2 ± 0.1
Rib eye area (cm^2^)	98.5 ± 0.8
Marbling score	508.8 ± 6.8
Intramuscular fat content (%)	5.03 ± 0.14
Fat thickness (mm)	17.6 ± 0.4
Yield grade	2.8 ± 0.1
Retail cut yield (%)	49.4 ± 0.1
Moisture content (%)	71.5 ± 0.1
Shear force (kg)	6.1 ± 0.1
Purge loss (%)	1.18 ± 0.03

### Rumen microbiota

The rumen microbiota was dominated by genera such as *Acidaminococcus*, *Lachnospiraceae* NK3A20 group, *Prevotella*, *Succiniclasticum*, and *Succinivibrionaceae* UCG-001 that are typically relatively abundant in the rumen of cattle (Henderson et al. 2015; Holman and Gzyl 2019) (Supplementary Table S1). The richness (number of ASVs) in the microbiota was 224.2 ± 3.0, and the diversity as measured by the inverse Simpson diversity index was 20.8 ± 0.7.

### Associations of carcass merit and meat quality attributes with specific bacterial genera

**Fig. 1:**
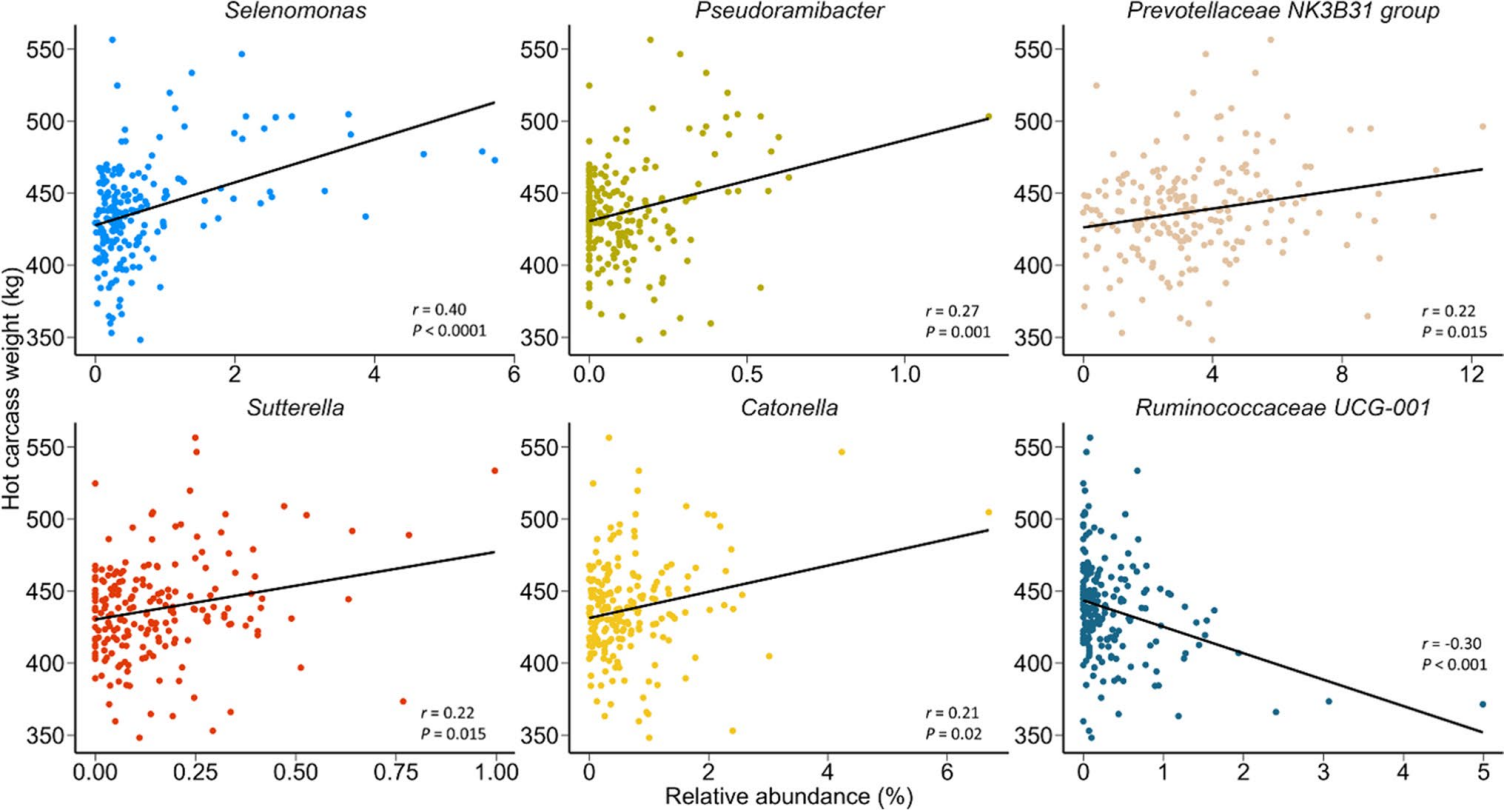
Scatter plots of the relative abundance (%) of genera vs. hot carcass weight. Only those genera with a Pearson correlation coefficient (*r*) of ≥ 0.20 and a *P* value of < 0.10 vs. hot carcass weight are included

Correlations between the relative abundance of bacterial genera with an overall relative abundance ≥ 0.1% (*n* = 38) and 12 different carcass merit and meat quality attributes (live weight, HCW, dressing percentage, REA, RCY, intramuscular fat content, marbling score, fat thickness, yield grade, moisture content, purge loss, and shear force) were assessed. The relative abundance of at least one genus was significantly (*P* < 0.10) correlated with live and HCW, dressing percentage, intramuscular fat content, marbling score, yield grade, moisture content, and purge loss. The highest correlations identified were between *Selenomonas* and live and HCW (Fig. 1; Supplementary Table S2; *r* ≥ 0.40; *P* < 0.0001). Hot carcass weight is determined after the head, hide, and internal organs have all been removed and so larger values are typically desirable. *Selenomonas* was also negatively correlated with purge loss (Supplementary Fig. S1), which is the loss of water from the meat during storage.

**Fig. 2:**
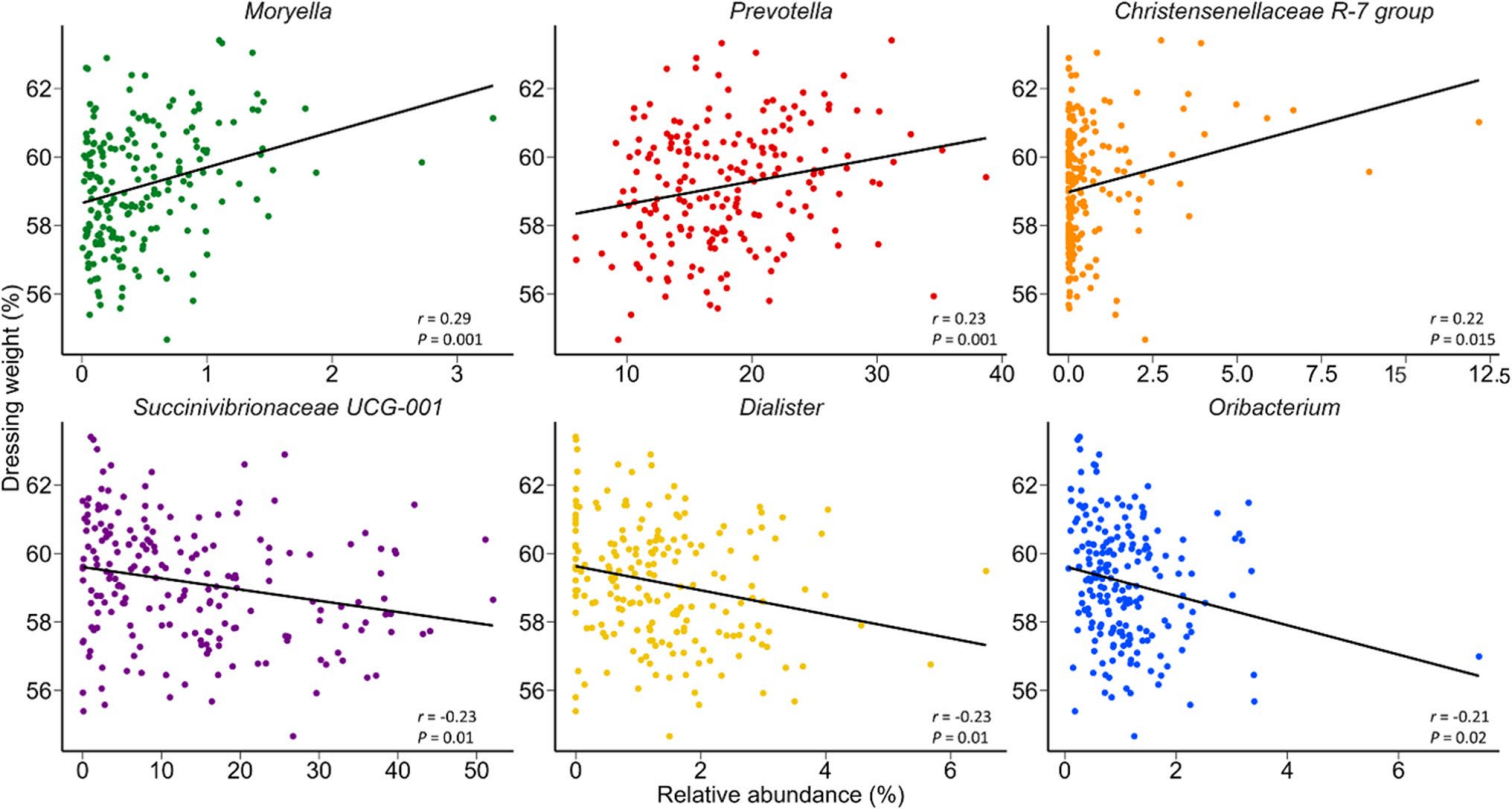
Scatter plots of the relative abundance (%) of genera in the rumen vs. dressing weight percentage. Only those genera with a Pearson correlation coefficient (*r*) of ≥ 0.20 and a *P* value of < 0.10 vs. dressing weight percentage are included

**Fig. 3:**
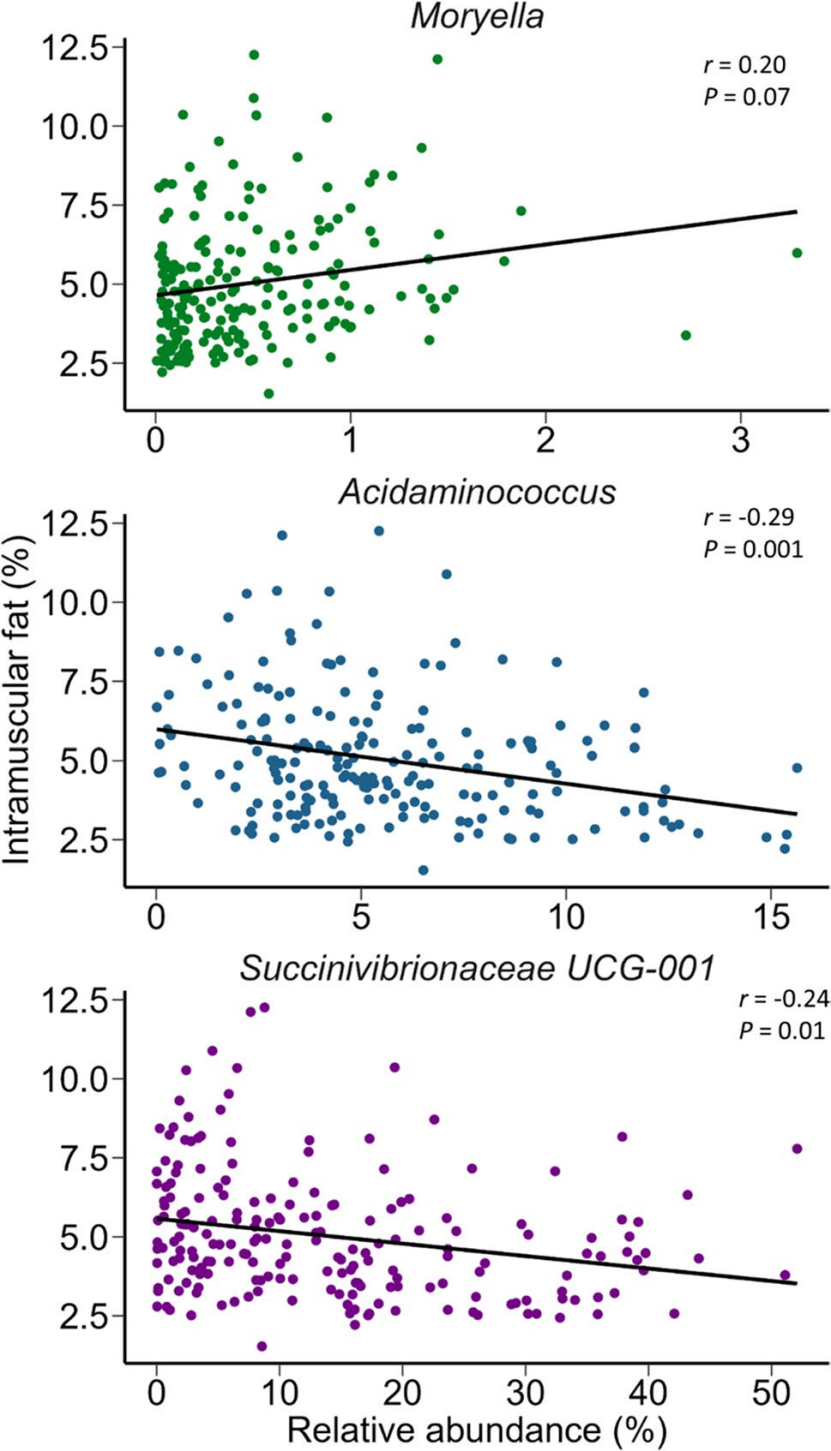
Scatter plots of the relative abundance (%) of genera in the rumen vs. intramuscular fat content (%). Only those genera with a Pearson correlation coefficient (*r*) of ≥ 0.20 and a *P* value of < 0.10 vs. intramuscular fat content are included

**Fig. 4:**
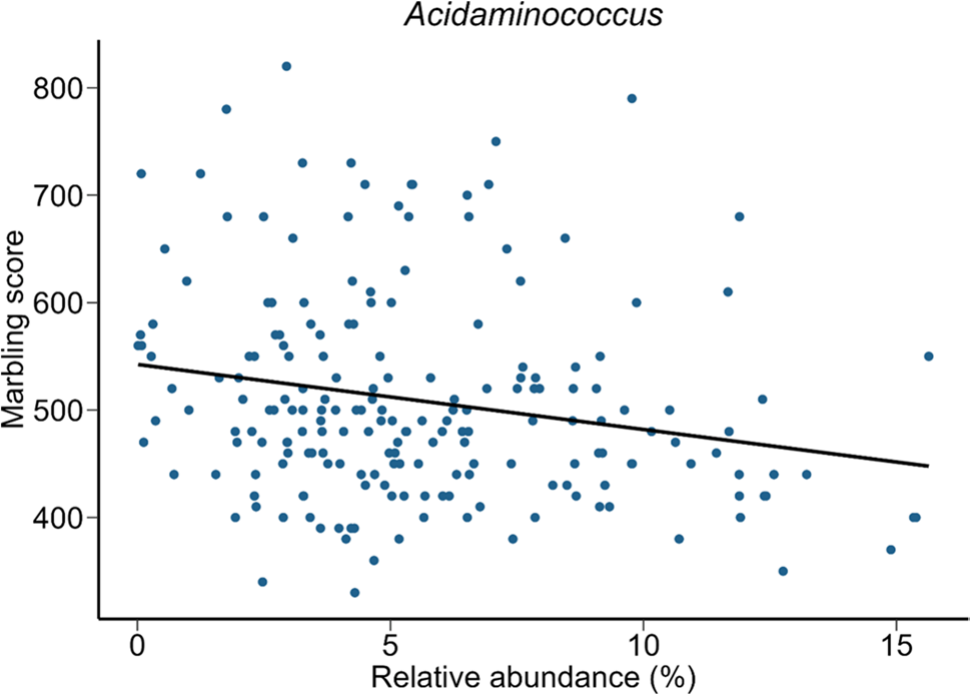
Scatter plot of the relative abundance (%) of *Acidaminococcus* in the rumen vs. marbling score

*Ruminococcaceae* UCG-001 was negatively correlated with HCW and live weight as well as the related dressing percentage. Dressing percentage is the HCW divided by the live weight prior to slaughter and thus is a good indicator of animal performance. Members of the *Christensenellaceae* R-7, *Moryella*, and *Prevotella* genera were significantly and positively correlated with dressing percentage (Fig. 2), although *Moryella* was negatively correlated with the live weight of the cattle prior to slaughter (Supplementary Fig. S2). The relative abundance of *Christensenellaceae* R-7, *Moryella*, and *Prevotella* was also positively correlated with the intramuscular fat content of the LT muscle (Fig. 3; Supplementary Table S2; *r* = 0.18 to 0.20; *P* < 0.10) while *Acidaminococcus* and *Succinivibrionaceae* UCG-001 were negatively correlated. *Acidaminococcus* also had the highest correlation (negative) with marbling score (Fig. 4). Members of the *Dialister* genus were likewise negatively correlated with marbling score (*r* = −0.18; *P* = 0.07) as well as dressing percentage (Fig. 2; *r* = -0.23; *P* = 0.01). In addition, *Acidaminococcus* and *Succinivibrionaceae* UCG-001 were positively correlated with moisture content (%) (Supplementary Fig. S3) while the relative abundance of *Prevotella* spp. was positively correlated with yield grade (*r* = 0.21) and purge loss (*r* = 0.26) (Supplementary Table S2; *P* < 0.10). There is a strong and negative correlation between fat and moisture content in meat (Pflanzer and de Felício 2011; Ueda et al. 2007), and so it is expected that those genera associated with lower fat content would also be linked with higher moisture content.

### Associations of carcass merit and meat quality attributes with rumen microbial community structure

**Fig. 5:**
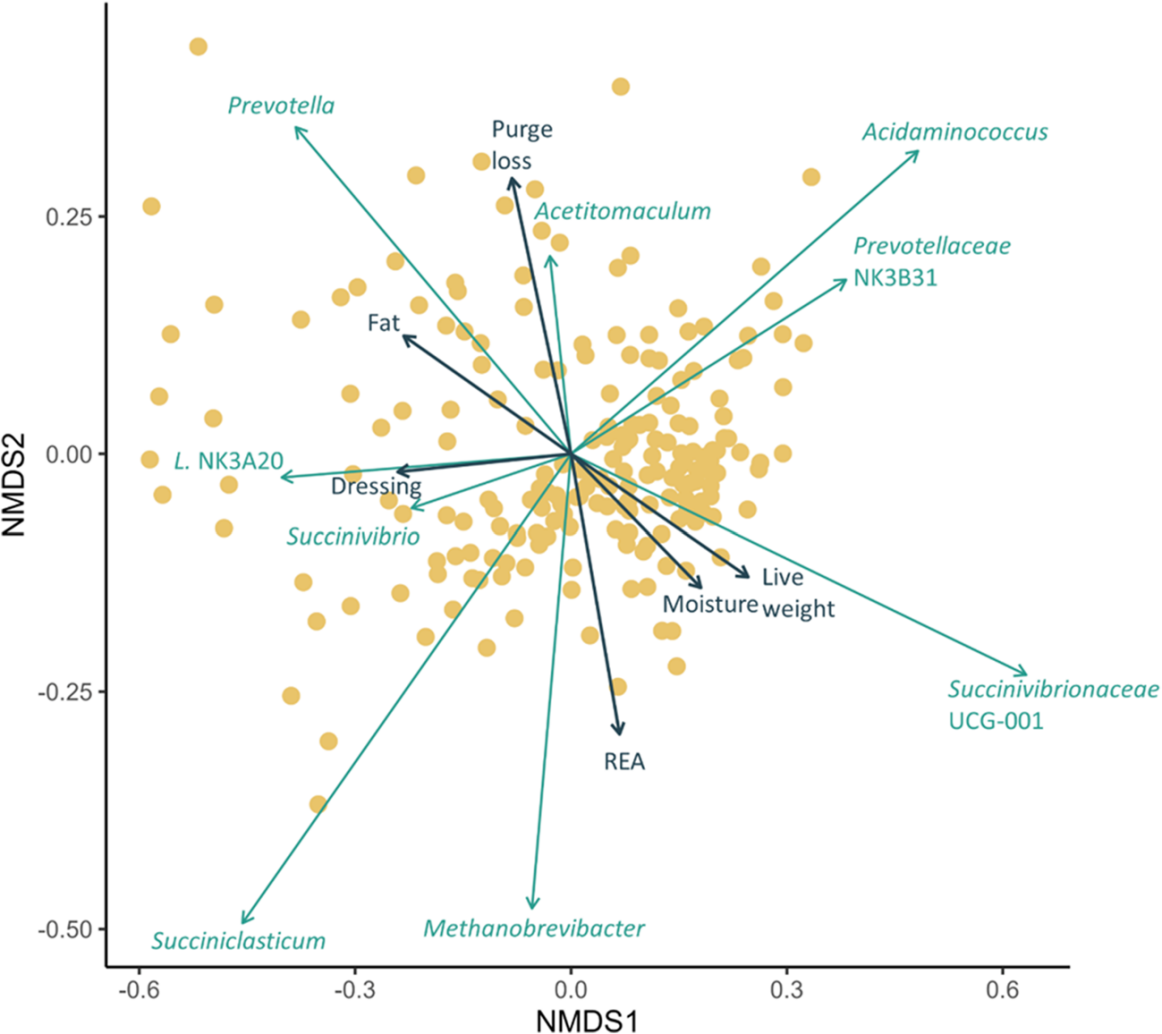
Non-metric multidimensional scaling (NMDS) plot of the Bray-Curtis dissimilarities of the rumen microbiota. Vectors based on the relative abundance of the ten relatively most abundant genera as well as carcass and meat quality attributes that have a statistically significant association (*P* < 0.05) with the ordinations are included. The vector length is proportional to the degree of correlation between the genus relative abundance as well as the carcass and meat quality attributes and the ordination. *L*. NK3A20: *Lachnospiraceae* NK3A20

The association of the 12 different carcass merit and meat quality attributes with the rumen microbial community structure was assessed using PERMANOVA and the Bray-Curtis dissimilarities. Although none of the attributes were strongly associated with the structure of the rumen microbiota, live weight (*R*^2^ = 0.01; *P* = 0.003), HCW (*R*^2^ = 0.01; *P* = 0.002), dressing percentage (*R*^2^ = 0.01; *P* = 0.002), REA (*R*^2^ = 0.01; *P* = 0.004), intramuscular fat content (*R*^2^ = 0.02; *P* = 0.0005), moisture (*R*^2^ = 0.01; *P* = 0.005), and purge loss (*R*^2^ = 0.01; *P* = 0.003) were all significant factors. Dressing percentage, intramuscular fat content, live weight, moisture content, purge loss, and REA were also significantly correlated with the NMDS ordination as were all but the [*Ruminococcus*] *gauvreauii* group among the ten relatively most abundant genera (Fig. 5).

### Low vs. high marbling scores

**Fig. 6:**
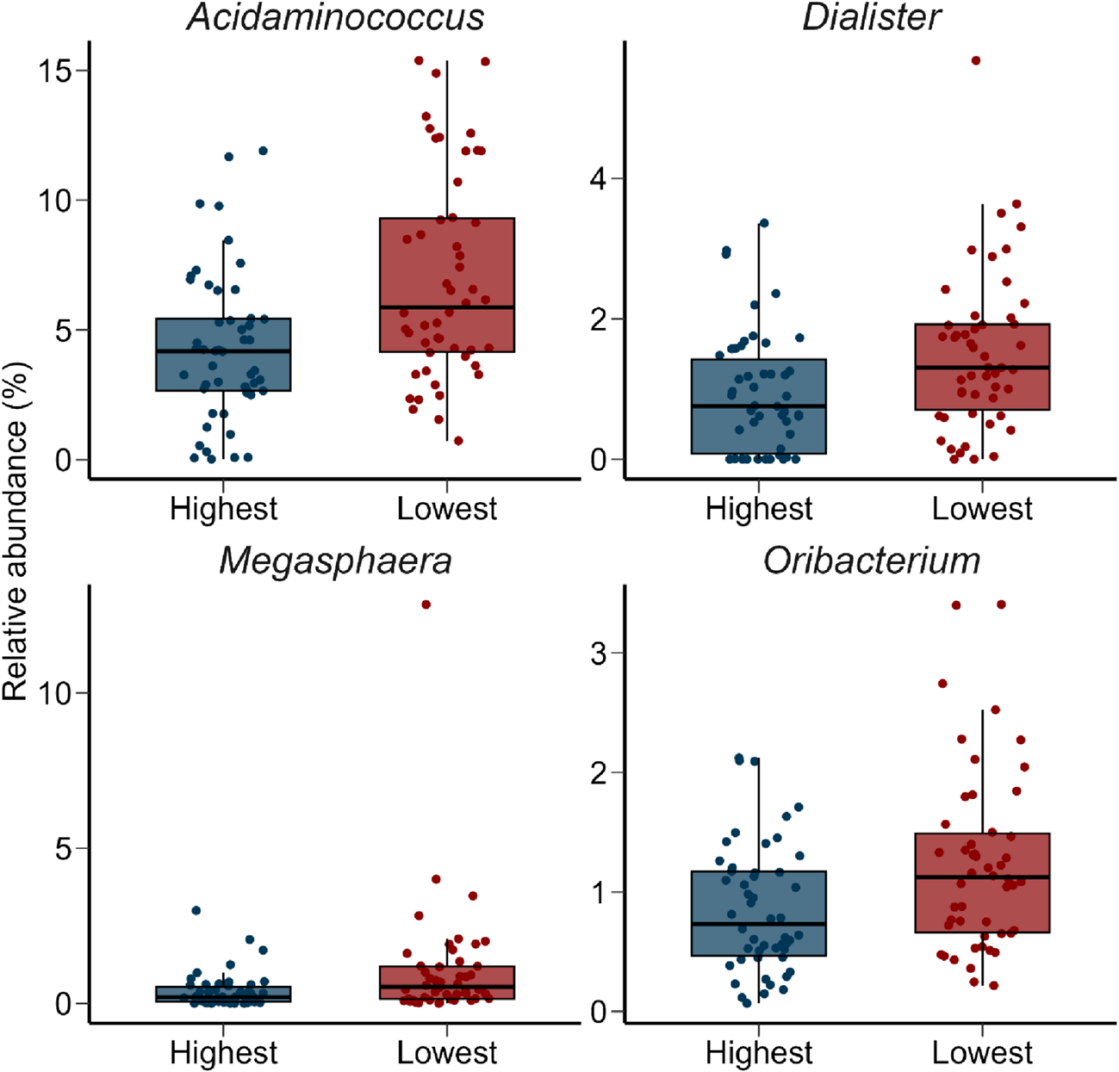
Box and whisker plots of the percent relative abundance of genera that were differentially abundant (*P* < 0.05) in the rumen microbiota between the cattle with the 50 highest and 50 lowest marbling scores. The interquartile range (IQR) (middle 50% of the data), the median value, and the whiskers representing 1.5 times the IQR are displayed

We also compared the rumen microbiota of animals whose carcasses had the 50 highest and 50 lowest marbling scores. In addition to having significantly higher marbling scores, the high marbling carcasses had significantly higher HCW, dressing percentage, intramuscular fat content, fat thickness, yield grade, and RCY percentage (Supplementary Table S3; *P* < 0.05). Although these two groups did not differ based on microbial community structure (PERMANOVA: *P* > 0.05, data not shown), four genera were relatively more abundant in the low marbling steers: *Acidaminococcus*, *Dialister*, *Megasphaera*, and *Oribacterium* (Fig. 6; *P* < 0.05). As with *Acidaminococcus* and *Dialister* spp., *Megasphaera* (*r* = −0.16) and *Oribacterium* spp. (*r* = −0.18) were also negatively correlated with intramuscular fat content (Supplementary Table S2). There were no significant differences between the high and low marbling score groups for any of the microbial diversity (inverse Simpson diversity index) or richness measures assessed (*P* > 0.05; data not shown).

## Discussion

Although the importance of the rumen microbiota in host nutrient and energy acquisition from nondigestible dietary carbohydrates is well-established, its impact on other traits, such as carcass merit and meat quality, remains less understood. Therefore, in this study, we characterized the rumen microbiota of 201 cattle just prior to slaughter and associated its composition with 12 different production, carcass merit, and meat quality traits. The relative abundance of at least one bacterial genus was found to be correlated with eight of the 12 traits assessed. Among these genera, *Selenomonas* had the strongest correlation as its relative abundance was positively correlated with both live and HCW and negatively correlated with purge loss.

Certain *Selenomonas* spp. (e.g., *Selenomonas ruminantium*) produce the SCFAs acetate and propionate from precursors such as lactate and succinate, and these SCFAs can then be used as energy by the host (Paynter and Elsden 1970). Propionate is also a major substrate for gluconeogenesis in ruminants (Aschenbach et al. 2010). Notably, *Selenomonas* spp. have been linked to increased marbling in Angus steers (Krause et al. 2020) and higher feed efficiency in dairy cattle (Xue et al. 2022). *Selenomonas* spp. in the rumen are also negatively associated with methane emissions (Smith et al. 2022). A study by Granja-Salcedo et al. (2019) reported a higher relative abundance of *Selenomonas* spp. and reduced methane emissions in grazing steers fed encapsulated nitrate. Thus, an increase in the abundance or proportion of *Selenomonas* spp. in the rumen may have multiple beneficial effects from a production standpoint.

Conversely, *Ruminococcaceae* UCG-001 was negatively correlated with live and HCW as well as dressing percentage. This uncultured group within the *Ruminococcaceae* family has also been associated with lower average daily gain in the rumen of feedlot steers (Daghio et al. 2021). Higher dressing percentage values were positively correlated with the relative abundance of *Christensenellaceae* R-7, *Moryella*, and *Prevotella* spp. Interestingly, higher intramuscular fat content and marbling scores in Angus steers (Krause et al. 2020) and higher feed efficiency in Charolais steers (Li et al. 2019) have previously been linked with the relative abundance of *Moryella* spp. Diets that are high in starch have been shown to increase the relative abundance of both *Moryella* and *Selenomonas* in the rumen of dairy cows (Darabighane et al. 2021). In our study, *Moryella* was also positively correlated with the intramuscular fat content. Presently, *Moryella indoligenes*, an acetate and butyrate producer, is the only described *Moryella* species (Carlier et al. 2007).

In addition to dressing percentage, the relative abundance of *Prevotella* spp. was positively correlated with intramuscular fat content, yield grade, and purge loss. *Prevotella* spp. are commonly among the most abundant bacteria in the rumen (Henderson et al. 2015; Holman and Gzyl 2019). However, *Prevotella* is a genetically and functionally diverse genus (Tett et al. 2021) that has recently been re-organized into several different genera (Hitch et al. 2022), and so the 16S rRNA gene sequences classified here as *Prevotella* may in fact now belong to multiple genera. *Christensenellaceae* R-7 represents an uncultured group of bacteria that were also positively correlated with intramuscular fat content. Similar to *M*. *indoligenes*, species in the *Christensenellaceae* family such as *Christensenella minuta* produce acetate and butyrate (Morotomi et al. 2012). As with propionate, acetate and butyrate both serve as energy sources for the host, and butyrate in particular is an important energy source for rumen epithelial cells (Remond et al. 1995). Acetate and butyrate are also lipogenic, and rumen concentrations of both SCFAs have been positively correlated with fat content of lamb (Wang et al. 2022). *Christensenellaceae* R-7 has also been reported to be enriched in the rumen microbiota of beef cattle fed high-fiber diets (Li et al. 2022).

As marbling score is one of the more important attributes that determine carcass value, we also compared the rumen microbiota of cattle whose carcasses had the 50 highest and 50 lowest marbling scores. *Acidaminococcus* spp., which were also negatively correlated with marbling score, were also the most differentially abundant between the highest and lowest marbling cattle. *Dialister*, *Megasphaera*, and *Oribacterium* were also relatively less abundant in the rumen microbiota of cattle with the lowest marbling scores. However, *Acidaminococcus* and *Dialister* spp. in the rumen have been positively associated with higher growth rates in beef cattle (Myer et al. 2015). Certain *Acidaminococcus* spp. strains can also produce acetate, butyrate, and propionate (Abdugheni et al. 2023; Jumas-Bilak et al. 2007), and so it is not immediately clear why these bacterial taxa would be associated with reduced fat content and marbling scores. *Oribacterium* spp. were relatively abundant overall (1.09% ± 0.06), and members of this genus have been linked with flax oil-supplemented diets in cattle (Huws et al. 2015). *Dialister* and *Megasphaera* are both classified within the *Veillonellaceae*, and this family has been reported to be associated with high residual feed intake (lower feed efficiency) in beef cattle (Li and Guan 2017).

In summary, our findings highlight significant correlations between various rumen microbial taxa such as *Acidaminococcus*, *Moryella*, and *Selenomonas* spp. and key carcass merit and meat quality attributes. Dietary strategies that alter the abundance of these taxa in the rumen may therefore improve certain beef carcass and meat quality attributes that are economically important. Finally, more work is needed to fully understand the association and relationship between the rumen microbiota and carcass merit and meat quality in beef cattle. Such information could be used to develop microbiome-targeted strategies aimed at improving animal production traits.

## Supplementary information

ESM 1 (PDF 731 kb)

## Data Availability

All 16S rRNA gene sequences are available under BioProject accession PRJNA949254.
