# Supplementary material for: Associations between the rumen microbiota and carcass merit and meat quality in beef cattle
Source: Appl Microbiol Biotechnol. 2024 Apr 6;108(1):287. doi: 10.1007/s00253-024-13126-1 (PMC10998782; doi:10.1007/s00253-024-13126-1)
Supplement: Supplementary file 1 — (PDF 731 kb) [file 253_2024_13126_MOESM1_ESM.pdf]

Supplementary material

Journal: Applied Microbiology and Biotechnology

Associations between the rumen microbiota and carcass merit and meat quality in beef cattle

Devin B. Holman[a]*, Katherine E. Gzyl[a], Haley Scott[a], Cara Service[a], Nuria Prieto[a], Óscar López-Campos[a]

[a]Agriculture and Agri-Food Canada, Lacombe Research and Development Centre, 6000 C&E Trail, Lacombe, AB, T4L1W1, Canada

*Correspondence:

Devin B. Holman

devin.holman@agr.gc.ca

Phone: 403-782-8143

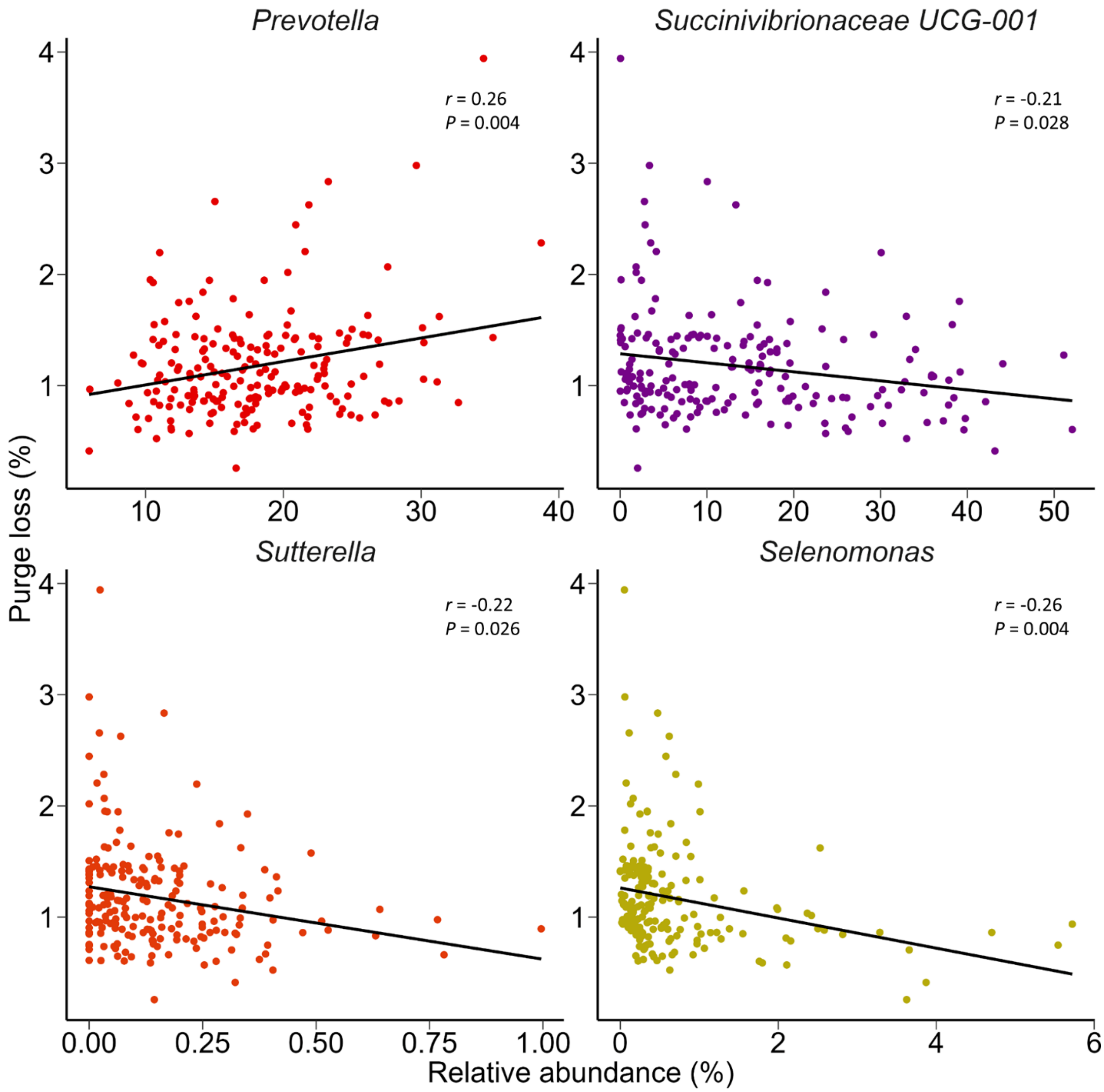

Supplementary Figure S1. Scatter plot of the percent relative abundance of genera in the rumen vs. meat purge loss.

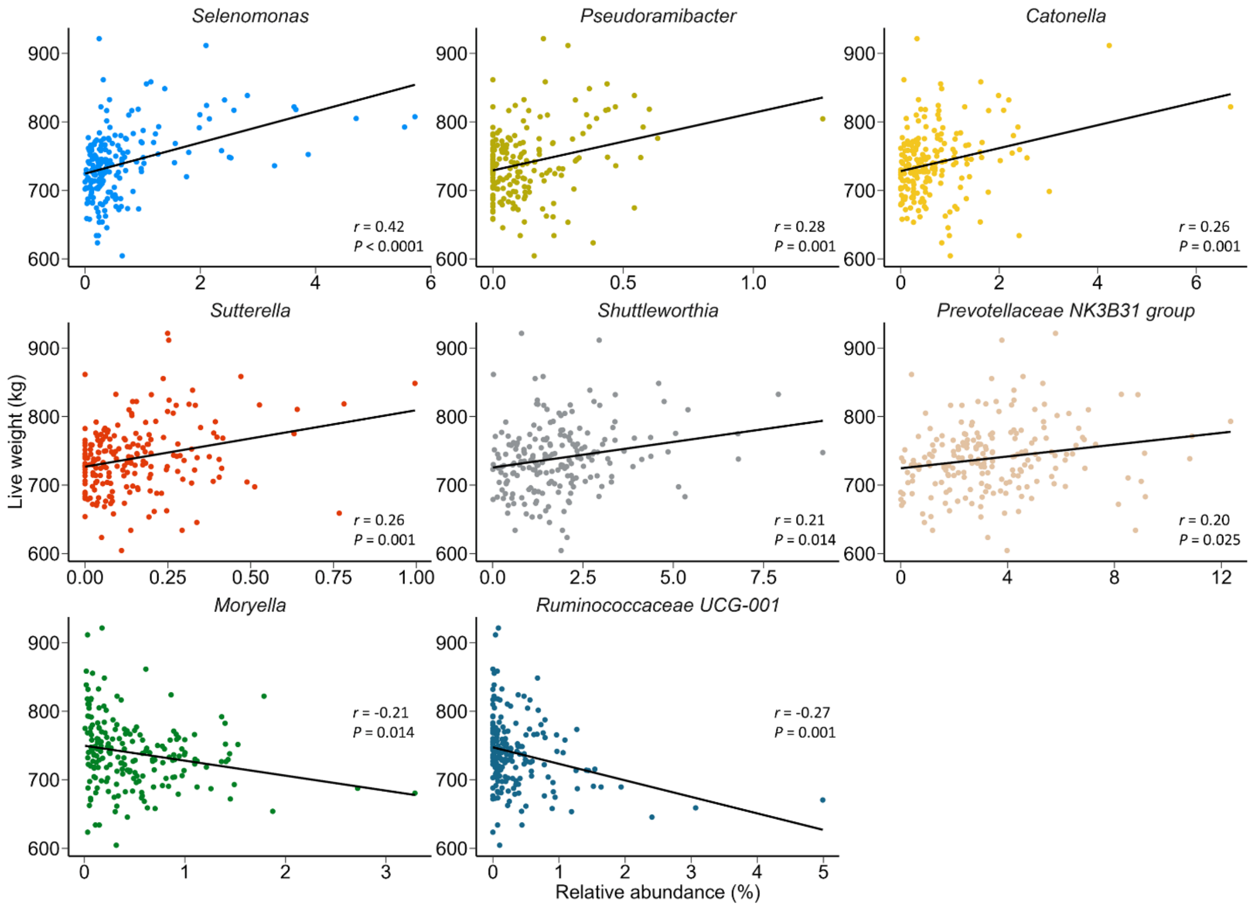

Supplementary Figure S2. Scatter plot of the percent relative abundance of genera in the rumen vs. the live weight of the cattle prior to slaughter.

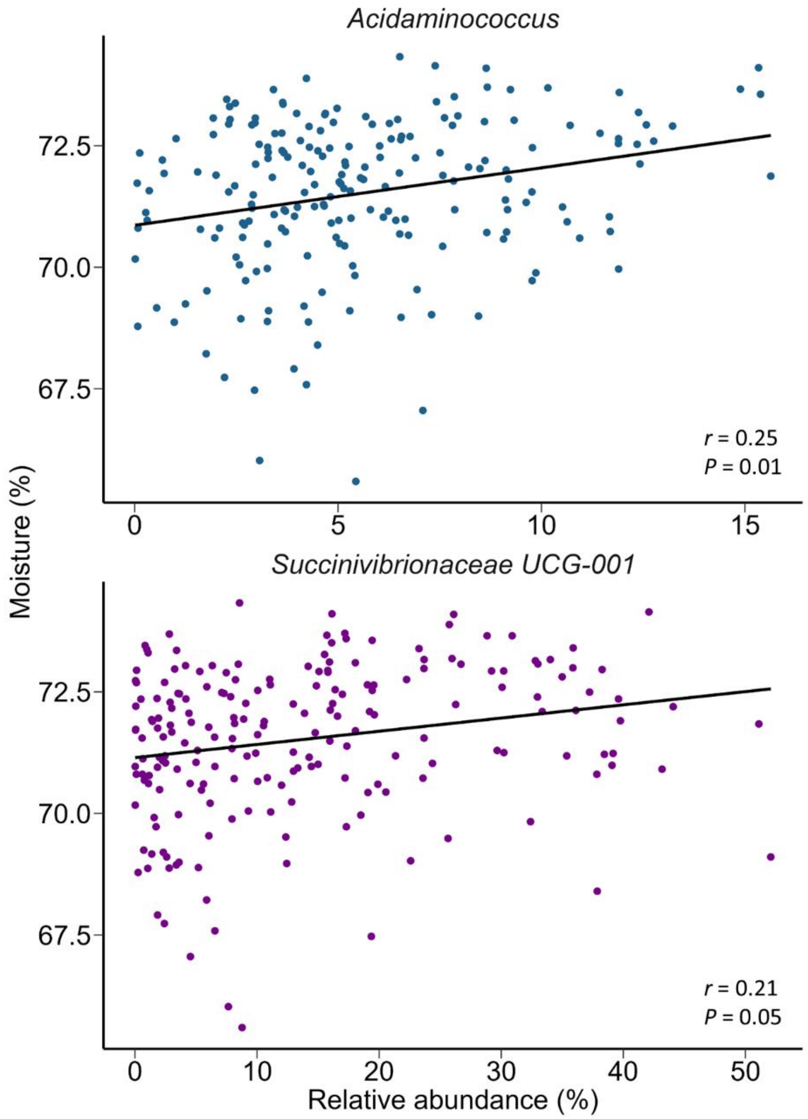

Supplementary Figure S3. Scatter plot of the percent relative abundance of genera in the rumen vs. the moisture content of the meat.

Supplementary Table S1. Archaeal and bacteria genera in the rumen microbiota with an overall percent relative abundance of greater than 0.1%.

**Genus**	**Relative abundance (%) ± SEM**
Prevotella	17.98 ± 0.41
Succinivibrionaceae UCG-001	13.78 ± 0.87
Succiniclasticum	8.60 ± 0.52
Acidaminococcus	5.57 ± 0.24
Lachnospiraceae NK3A20 group	4.44 ± 0.15
Prevotellaceae NK3B31 group	3.41 ± 0.16
Methanobrevibacter	3.40 ± 0.13
[Ruminococcus] gauvreauii group	3.00 ± 0.12
Acetitomaculum	2.24 ± 0.18
Succinivibrio	2.09 ± 0.25
Shuttleworthia	1.83 ± 0.10
Ruminococcus	1.38 ± 0.08
Dialister	1.35 ± 0.08
Oribacterium	1.09 ± 0.06
Rikenellaceae RC9 gut group	1.01 ± 0.07
Ruminococcaceae NK4A214 group	0.97 ± 0.04
Prevotellaceae UCG-001	0.72 ± 0.07
Christensenellaceae R-7 group	0.67 ± 0.10
Catonella	0.66 ± 0.05
Selenomonas	0.65 ± 0.06
Megasphaera	0.63 ± 0.08

Olsenella	0.62 ± 0.05
Moryella	0.48 ± 0.03
Syntrophococcus	0.43 ± 0.02
Ruminococcaceae UCG-001	0.34 ± 0.04
[*Eubacterium*] *nodatum* group	0.33 ± 0.02
Mogibacterium	0.30 ± 0.01
Mitsuokella	0.29 ± 0.03
Pyramidobacter	0.26 ± 0.02
Agathobacter	0.23 ± 0.02
Treponema	0.23 ± 0.03
Ruminococcaceae UCG-002	0.15 ± 0.01
Sutterella	0.15 ± 0.01
Coprococcus	0.14 ± 0.01
Pseudoramibacter	0.12 ± 0.01
[*Eubacterium*] *ruminantium* group	0.12 ± 0.01
Ruminococcaceae UCG-005	0.11 ± 0.02
Lachnospiraceae XPB1014 group	0.11 ± 0.01

Supplementary Table S2. Correlations between carcass merit/quality attributes and bacterial genera. Only those correlations with a Benjamini-Hochberg corrected *P* value of < 0.10 are included.

Attribute	Correlation coefficient (r)	P-value
**Live weight**		
Selenomonas	0.4157	0.0000
Pseudoramibacter	0.2756	0.0014
Catonella	0.2618	0.0014
Sutterella	0.2611	0.0014
Shuttleworthia	0.2141	0.0143
Prevotellaceae NK3B31 group	0.1967	0.0249
Mitsuokella	0.1752	0.0479
Succinivibrionaceae UCG-001	0.1738	0.0479
[*Ruminococcus*] *gauvreauii* group	-0.1862	0.0350
Moryella	-0.2116	0.0143
Ruminococcaceae UCG-001	-0.2681	0.0014
**Hot carcass weight**		
Selenomonas	0.3983	0.0000
Pseudoramibacter	0.2690	0.0014
Prevotellaceae NK3B31 group	0.2176	0.0146
Sutterella	0.2176	0.0146
Catonella	0.2071	0.0201
Oribacterium	-0.1794	0.0514
[*Ruminococcus*] *gauvreauii* group	-0.1862	0.0441
Ruminococcaceae UCG-001	-0.2974	0.0003
**Dressing weight (%)**		
Moryella	0.2894	0.0012
Prevotella	0.2274	0.0107
Christensenellaceae R-7 group	0.2244	0.0107
Mitsuokella	-0.1893	0.0345
Ruminococcaceae UCG-001	-0.1940	0.0321
Oribacterium	-0.2067	0.0210
Dialister	-0.2273	0.0107
Succinivibrionaceae UCG-001	-0.2314	0.0107
**Marbling**		
Acidaminococcus	-0.2122	0.0945
**Fat content**		
Moryella	0.1955	0.0664
Prevotella	0.1877	0.0664
Christensenellaceae R-7 group	0.1757	0.0685
Coprococcus	0.1681	0.0724
Lachnospiraceae NK3A20 group	0.1680	0.0724

Megasphaera	-0.1575	0.0884
[*Ruminococcus*] *gauvreauii* group	-0.1635	0.0773
Oribacterium	-0.1767	0.0685
Dialister	-0.1845	0.0664
Succinivibrionaceae UCG-001	-0.2442	0.0091
Acidaminococcus	-0.2902	0.0011
**Yield grade**		
Prevotella	0.2078	0.0800
Pseudoramibacter	0.2011	0.0800
**Moisture (%)**		
Acidaminococcus	0.2529	0.0117
Succinivibrionaceae UCG-001	0.2122	0.0500
**Purge loss**		
Prevotella	0.2586	0.0040
Succinivibrionaceae UCG-001	-0.2089	0.0277
Sutterella	-0.2159	0.0264
Selenomonas	-0.2589	0.0040

Supplementary Table S3. Carcass merit and meat quality traits of the carcasses with the 50 highest and 50 lowest marbling scores. *P* values are from the Mann–Whitney U test.

**Attribute**	**High marbling**	**Low marbling**	**P-value**
Live weight (kg)	739.2 ± 8.0	728.2 ± 7.4	0.79
Hot carcass weight (kg)	443.8 ± 5.3	426.0 ± 4.8	**0.03**
Hot dressing (%)	60.0 ± 0.2	58.5 ± 0.2	**9.89E-06**
Rib eye area (cm2)	97.4 ± 1.6	98.9 ± 1.6	0.46
Marbling score	645.8 ± 9.7	406.8 ± 3.7	**< 2.2E-16**
Intramuscular fat content (%)	7.31 ± 0.26	3.32 ± 0.10	**< 2.2e-16**
Fat thickness (mm)	20.7 ± 0.9	15.2 ± 0.7	**1.07E-05**
Yield grade	3.2 ± 0.1	2.5 ± 0.1	**1.32E-05**
Retail cut yield (%)	48.4 ± 0.3	50.0 ± 0.3	**4.13E-05**
Moisture content	69.7 ± 0.2	72.8 ± 0.1	**< 2.2e-16**
Shearing	5.9 ± 0.2	6.24 ± 0.2	0.28
Purge loss	1.16 ± 0.07	1.22 ± 0.07	0.31
